# Supplementary material for: Contrast-enhanced and indirect computed tomography lymphangiography accurately identifies the cervical lymphocenter at risk for metastasis in pet dogs with spontaneously occurring oral neoplasia
Source: PLoS One. 2023 Mar 2;18(3):e0282500. doi: 10.1371/journal.pone.0282500 (PMC9980747; doi:10.1371/journal.pone.0282500)
Supplement: S1 Table — (DOCX) [file pone.0282500.s001.docx]

**S1 Table. Clinical characteristics of the 39 dogs with oral tumors enrolled**.

| Age (years) | |
| --- | --- |
| Mean ± standard deviation | 11.1 ± 2.4 |
| Sex | Number of dogs (%) |
| Spayed female | 18 (46%) |
| Neutered male | 19 (49%) |
| Intact male | 2 (5%) |
| Weight (kg) | |
| Median | 25.9 |
| Range | 7.2-43.2 |
| Breed | Number of dogs (%) |
| Mixed breed | 7 (18%) |
| Purebred | 32 (82%) |
| Rottweiler | 2 |
| Rat Terrier | 2 |
| Irish Setter | 1 |
| Pug | 1 |
| Labrador Retriever | 4 |
| Golden Retriever | 3 |
| German Shepherd Dog | 2 |
| Italian Spinone | 2 |
| Cocker Spaniel | 2 |
| Husky | 2 |
| German Short Haired Pointer | 2 |
| Australian Cattle Dog | 1 |
| Bulldog | 1 |
