# Supplementary material for: Contrast-enhanced and indirect computed tomography lymphangiography accurately identifies the cervical lymphocenter at risk for metastasis in pet dogs with spontaneously occurring oral neoplasia
Source: PLoS One. 2023 Mar 2;18(3):e0282500. doi: 10.1371/journal.pone.0282500 (PMC9980747; doi:10.1371/journal.pone.0282500)
Supplement: S2 Table — Significant values are shaded. When no data reported logistic regression failed. (DOCX) [file pone.0282500.s002.docx]

**S2 Table. Significance of logistic regression model and receiver operator characteristic (ROC) curve analysis for post-contrast and ICTL features of each mandibular lymph nodes (MLN) and medial retropharyngeal lymph node (MRLN) category separately from 39 dogs with oral tumors.**

| **Characteristic** | **R medial**  **MLN** | **R lateral MLN** | **R MRLN** | **L medial MLN** | **L lateral MLN** | **L MRLN** |
| --- | --- | --- | --- | --- | --- | --- |
| LN metastasis  Number  Percentage | 2  5.1% | 11  28.2% | 3  7.7% | 7  17.9% | 6  15.4% | 1  2.6% |
| Short axis | AUC: 0.93  P=0.006 | AUC: 0.86  P=0.01 | AUC: 0.90  P=0.004 | AUC: 0.85  P<0.001 | AUC:0.77  P=0.01 | - |
| Long axis | AUC: 0.96  P=0.003 | AUC: 0.63  P=0.08 | AUC: 0.77  P=0.04 | AUC: 0.90  P<0.001 | AUC: 0.66  P=0.08 | - |
| Shape score | AUC: 0.61  P=0.5 | AUC: 0.71  P=0.15 | AUC: 0.60  P=0.42 | AUC: 0.79  P=0.004 | AUC: 0.70  P=0.07 |  |
| Contrast score | AUC: 0.61  P=0.5 | AUC: 0.71  P=0.15 | AUC: 0.68  P=0.22 | AUC: 0.67  P=0.10 | AUC: 0.70  P=0.07 |  |
| Subjective score | AUC: 0.68  P=0.18 | AUC: 0.80  P=0.001 | AUC: 0.78  P=0.17 | AUC: 0.79  P=0.002 | AUC: 0.74  P=0.01 |  |
| CT score A | AUC: 0.70  P=0.28 | AUC: 0.68  P=0.13 | AUC: 0.72  P=0.19 | AUC: 0.82  P=0.003 | AUC: 0.69  P=0.08 | - |
| CT score B | AUC: 0.72  P=0.21 | AUC: 0.7  P=0.04 | AUC: 0.76  P=0.07 | AUC: 0.86  P=0.001 | AUC: 0.74  P=0.03 |  |
| ITCL contrast score |  |  | AUC: 0.60  P=0.37 | AUC: 0.61  P=0.25 | AUC: 0.59  P=0.39 | - |
| Lymphangiography  Score | AUC: 0.72  P=0.24 | AUC: 0.66  P=0.23 | AUC: 0.49  P=0.87 | AUC: 0.57  P=0.46 | AUC: 0.61  P=0.38 | - |

Significant values are shaded. When no data reported logistic regression failed.
