# Supplementary material for: Contrast-enhanced and indirect computed tomography lymphangiography accurately identifies the cervical lymphocenter at risk for metastasis in pet dogs with spontaneously occurring oral neoplasia
Source: PLoS One. 2023 Mar 2;18(3):e0282500. doi: 10.1371/journal.pone.0282500 (PMC9980747; doi:10.1371/journal.pone.0282500)
Supplement: Attachment — Submitted filename: Response to reviewers.docx [file pone.0282500.s003.docx]

The authors want to thank the editor and reviewers for their suggestions, please see specific responses below:

**Response to Editor**

When submitting your revision, we need you to address these additional requirements.

1. Please ensure that your manuscript meets PLOS ONE's style requirements, including those for file naming. The PLOS ONE style templates can be found at
   <https://journals.plos.org/plosone/s/file?id=wjVg/PLOSOne_formatting_sample_main_body.pdf> and 
   <https://journals.plos.org/plosone/s/file?id=ba62/PLOSOne_formatting_sample_title_authors_affiliations.pdf>

**Response:** The manuscript has been checked and the formatting has been updated where there were oversights. We have also correctly named the files and re-uploaded; we apologize for this oversight.

2. Thank you for stating the following in the Acknowledgments Section of your manuscript: "The authors thank the University of Minnesota Grant in Aid Program for funding this work (awarded to SG [PI], JL and CO)." We note that you have provided funding information that is not currently declared in your Funding Statement. However, funding information should not appear in the Acknowledgments section or other areas of your manuscript. We will only publish funding information present in the Funding Statement section of the online submission form.
   Please remove any funding-related text from the manuscript and let us know how you would like to update your Funding Statement. Currently, your Funding Statement reads as follows:

"University of Minnesota Grant in Aid
PI: SG, CO-I: JL,CO,NS
Grant #:  1801 - 11652 - 20562 - 4214572
https://research.umn.edu/funding-awards/grant-aid

The funders had no role in study design, data collection and analysis, decision to publish, or preparation of the manuscript."

Please include your amended statements within your cover letter; we will change the online submission form on your behalf.

**Response:** The submitted Funding Statement is correct. We have removed the redundant wording from our Acknowledgements section; we apologize for this oversight in our initial version. No additional changes to the statement are required.

3. Please review your reference list to ensure that it is complete and correct. If you have cited papers that have been retracted, please include the rationale for doing so in the manuscript text, or remove these references and replace them with relevant current references. Any changes to the reference list should be mentioned in the rebuttal letter that accompanies your revised manuscript. If you need to cite a retracted article, indicate the article’s retracted status in the References list and also include a citation and full reference for the retraction notice.

**Response:** The authors have checked the references and are not aware of any retracted papers cited in our manuscript. EndNote20 automatically checks references for retractions and updates each time a library is opened. We are aware that no software is perfect for retraction watches. Please let us know if we have been unable to locate a retracted article flagged by the Journal. We have updated our reference list in our revised manuscript as we have addressed comments by the reviewers and have highlighted this in the cover letter.

**Response to Reviewer 1**

1. inclusion/exclusion criteria should be better clarified by extending their description.

**Response:** This has been updated

2. Discussions can be expanded and improved by citing relevant articles (I suggest authors to read and insert in references the following article PMID: 33400886)
   Considered all this points, I think it could be of interest for the readers and, in my opinion, it deserves the priority to be published after minor revisions.

**Response:** We appreciate the suggestions and have amended the discussion to include specific key points from the breast carcinoma literature and have applied this concept to our canine populations as well.

**Response to Reviewer 2**

1. There are several abbreviations that are used in the introduction that are only used infrequently in the remainder of the manuscript which occasionally confuse more than clarify. Recommend reassessing the use of FN, pN+, pN0 as these terms aren’t used that frequently in the manuscript. Also, in line 79, consider removing “(cN0)” as this is just a bit confusing when “cN0 neck” is used the remainder of the time.

**Response:** Thank you for this suggestion, we have written out these abbreviations to improve clarity. We have elected to keep pN0 and pN+ in the body of the manuscript for completeness and to encourage this language to be utilized more frequently in veterinary medicine. Please advise, if you feel strongly about removing them all together. We have also added neck to areas where we use cN0 or cN+

2. Page 4, line 91-94: Consider citing the actual NCCN guidelines here. Also, the NCCN guidelines have been updated since 2014 – please confirm that this statement is still accurate.
   **Response:** We appreciate the note that the NCCN guidelines are routinely updated. We have referenced the current NCCN guidelines for head and neck cancer and altered our wording to correctly reflect the NCCN recommendations.
3. Page 6, lines 145-146: While the previous literature is references, consider confirming here that the injections were performed peritumorally in four quadrants (if that what was done) just to clarify that the number of sites is not the minor modification that was made to the previous published procedure.

**Response:** Thank you for noting this, we have updated to clarify that we also did 4-site peritumoral injections.

4. Page 7, lines 166-167; Table 1; Fig 2: Please provide a bit more detail as to how the subjective score for metastasis was determined.

**Response:** This has been updated.

5. Page 11, Table 3: It’s a bit confusing why two dogs with APOT were included in this study. It’s clear that for one dog the original histo was SCC, but it’s not clear why the 2nd dog with APOT was enrolled. It’s clear from the published case report that CT lymphography was performed on this case (at the same time the CT of the thorax was performed), but it is unclear as to how it originally met the inclusion for this study. Please clarify. If this dog did not meet the enrollment criteria for this study, it should be removed from analysis.

**Response:** This dog was included due to the biologically aggressive nature of the tumor and the presence of multifocal pulmonary metastasis. We have clarified the inclusion criteria in our methods to reflect our desire to include variable clinical scenarios by which dogs with biologically aggressive tumors were included.

6. Page 19, lines 362-363: pN+ is used infrequently in this manuscript; consider just stating, “…metastasis was identified in the cervical basin using histopathology,…”

**Response:** This has been modified slightly, we have still left in the pN+ as the comparative oncology, dentistry and oral surgery, and pathology groups would like to encourage the routine use of this terminology in veterinary medicine. Should the reviewer and editor want this to be removed in order to be published, we will defer and alter the wording.

7. Figures 2 & 3: Resolution is poor for both these figures – difficult to evaluate due to imagine quality.

**Response:** All Images have been updated to increase resolution, and the file names have been changed to ensure they meet PLOS ONE authorship guidelines
